# Saikosaponin-d Suppresses COX2 Through p-STAT3/C/EBPβ Signaling Pathway in Liver Cancer: A Novel Mechanism of Action

**DOI:** 10.3389/fphar.2019.00623

**Published:** 2019-05-29

**Authors:** Mudan Ren, Eileen McGowan, Yarui Li, Xiaofeng Zhu, Xinlan Lu, Zhanfang Zhu, Yiguang Lin, Shuixiang He

**Affiliations:** ^1^Department of Gastroenterology, The First Affiliated Hospital of Xi’an Jiao tong University, Xi’an, China; ^2^School of Life Sciences, University of Technology Sydney, Broadway, NSW, Australia; ^3^Department of Surgery, First Affiliated Hospital of Sun Yat-sen University, Guangzhou, China

**Keywords:** saikosaponins, hepatocellular carcinoma, signaling pathways, COX-2, AG490, apoptosis, HepG2, SMMC-7721

## Abstract

Saikosaponin-d (SSd) is an active extract from *Radix Bupleuri*, the dried root from the plant *Bupleurum falcatum* used in China for thousands of years to treat liver diseases. The SSd extract possesses valuable pharmacological activities including anti-cancer and anti-inflammatory effects; however, the mechanism underlying the anti-cancer activity of SSd is largely unknown. Here, we explored the mechanism of action of SSd as an anti-cancer agent for liver cancer in two human hepatocellular carcinoma cell lines. Using MTT and annexin-V-FITC/PI assays, Western blots, immunohistochemistry, qRT-PCR, luciferase reporter assay, and a JAK2-specific inhibitor (AG490), we demonstrated that the anti-tumorigenic effects of SSd act through the intermediatory p-STAT3/C/EBPβ signaling pathway to suppress cyclooxygenase (COX)-2. SSd effectively inhibited cell proliferation in a dose-dependent manner. Apoptosis was significantly increased in cells treated with SSd (2.5–15 µg/ml) with concurrent increase and decrease in pro- and anti-apoptosis proteins, respectively. COX-2, C/EBPβ, and p-STAT3 were significantly decreased, at both the translational and transcriptional levels, by SSd treatment. AG490 produced similar inhibitory effects on STAT3, p-STAT3, C/EBPβ, and COX-2. In conclusion, our data suggest that SSd controls liver cancer proliferation through suppression of the p-STAT3/C/EBPβ signaling pathway inhibiting COX2 expression. These findings further our understanding of the pharmacological action of SSd, providing new information on SSd mechanism of action and showing potential for SSd as a novel therapy for liver cancer.

## Introduction

Hepatocellular carcinoma (HCC) is the fifth most common malignancy and the second cancer killer worldwide (Stewart and Wild, 2017). Incidence and mortality rates of HCC are most prevalent in eastern and southeastern Asia (Stewart and Wild, 2017). HCC is aggressive and has a poor prognosis, with an overall ratio of mortality to incidence of 0.95. The majority of HCC patients are diagnosed at an advanced stage when treatment options are very limited and mostly ineffective. Therefore, new effective therapeutic strategies are needed to improve long-term survival. Saikosaponin-d (SSd) is a natural plant product and has been proposed as a new efficacious treatment for HCC patients (Xu et al., 2016; Yang et al., 2017; Yuan et al., 2017).

For thousands of years, herbs have been used in traditional Chinese medicine (TCM) to treat various liver diseases, including cancer (Yang et al., 2017; Yuan et al., 2017). *Radix Bupleuri* is a popular herb that is still used today in about 150 traditional Chinese prescriptions for various clinical conditions including liver diseases in China (Xie et al., 2009; Yang et al., 2017; Yuan et al., 2017). *Radix Bupleuri* (Chaihu in Chinese, Saiko in Japanese) is the dried root of the plant *Bupleurum falcatum L* (Yang et al., 2017; Yuan et al., 2017) and is commonly used as a principal herb in a classic compound herbal formula called *Xiao Chai Hu Tang* (XCHT, or *Sho-saiko-to* in Japanese) to treat HCC (Oka et al., 1995; Shimizu, 2000; Zheng et al., 2013). In a prospective randomized clinical trial, Oka et al. (1995)convincingly showed that XCHT prevented the development of HCC in patients with cirrhosis.

The phytochemistry, pharmacology, and mode of action of the genus *Bupleurum* (Ashour and Wink, 2011) and the derivatives of the dried root, *Radix Bupleuri*, have been extensively characterized (Bao et al., 2004). The active saikosaponins and extracts isolated from *Radix Bupleuri* and their applications have been recently reviewed (Yuan et al., 2017). SSd is one of the major active triterpene saponins, a natural molecule extracted from *Radix Bupleuri*. Pharmacological benefits of SSd include anti-cancer, anti-inflammatory, antipyretic, antimicrobial, antiviral, hepato-protective, and immunomodulatory effects (Yuan et al., 2017). Since the anti-cancer properties of SSd were first identified in 1994 (Motoo and Sawabu, 1994), anti-proliferation, anti-metastasis, and anti-angiogenesis have been demonstrated both *in vitro* (Motoo and Sawabu, 1994; Hsu et al., 2004) and *in vivo* (Oka et al., 1995; Lu et al., 2012; Xu et al., 2016). The *in vitro* antitumor properties of SSd have been demonstrated in human hepatoma (Motoo and Sawabu, 1994), human hepatocellular cells (He et al., 2014) (SMMC7721, HepG2, Hep3B, and 2.2.15), lung cancer, A549 cells (Hsu et al., 2004), prostate carcinoma, DU145 cells (Yao et al., 2014), cervical carcinoma, Hela cells (Wong et al., 2013), breast carcinoma, MCF-7 cells (Wang et al., 2010), and thyroid cancer cells (ARO, 8305C, and SW1736) (Liu and Li, 2014). However, the exact mechanisms by which SSd exerts its anti-cancer effects are unclear.

COX-2 is a rate-limiting enzyme in the production of prostaglandins promoted by a variety of factors including cytokines, growth factors, and tumor promoters (Vane et al., 1998). The overexpression of COX-2 is observed in many human cancers such as prostate (Gupta et al., 2000), breast (Singh et al., 2005), lung (Hida et al., 1998), and liver cancer (Bae et al., 2001; Hu et al., 2003; Leng et al., 2003; Foster et al., 2007; Schmitz et al., 2009; Guo et al., 2015; Yang et al., 2016; Chen et al., 2017). The importance of the strong association between COX-2 overexpression and HCC has been well documented (Bae et al., 2001; Hu et al., 2003; Leng et al., 2003; Cervello and Montalto, 2006; Foster et al., 2007; Schmitz et al., 2009; Guo et al., 2015; Yang et al., 2016; Chen et al., 2017). Several studies found that COX-2 promoted HCC cell growth, migration, and invasion (Leng et al., 2003; Guo et al., 2015). In HCC patients, the protein expression of COX-2 correlates well with differentiation grades, suggesting that abnormal COX-2 expression has an important effect in hepatocarcinogenesis (Bae et al., 2001). Recently, *in vivo* mouse studies demonstrated that overexpression of COX-2 in the liver was sufficient to induce HCC (Chen et al., 2017). COX-2 overexpression has been shown to promote tumor initiation and proliferation and inhibit apoptosis by mediating the activation of downstream oncogenic pathways (Sobolewski et al., 2010). Thus, the role of COX-2 in the pathogenesis of HCC is relatively well defined, and deregulation of the COX-2 signaling pathway may serve as a basis for designing novel-targeted therapeutic strategies for cancer therapy. What is unclear is the upstream regulatory network controlling COX-2 expression.

Our laboratory has played an important role in describing the significance of SSd suppression of COX-2 in HCCs and the SSd’s chemo-preventive effect on liver cancer associated with COX-2 inhibition (He et al., 2006;He et al., 2014; Liang et al., 2010; Lu et al., 2012). In this study, we extended these findings to understand the upstream mechanism of COX-2 inhibition by SSd treatment. The transcription factor CCAAT/enhancer binding protein β (C/EBPβ) is one of the key regulators implicated in COX-2 expression (Straccia et al., 2013). Herein, for the first time, we presented our latest data demonstrating how SSd acted through the p-STAT3/C/EBPβ signaling pathway, leading to COX-2 suppression and antitumor activity in human HCC cells. This information will contribute to our new understanding of the mechanisms of action by which SSd contributes to the treatment and prevention of HCC.

## Materials and Methods

### Reagents and Chemicals

RPMI-1640 medium was purchased from Invitrogen Life Technologies, CA, USA. Fetal bovine serum (FBS) was supplied by HyClone, UT, USA. Tyrphostin AG490 (a JAK2 kinase inhibitor), dimethyl sulfoxide (DMSO), and acetic acid were purchased from Sigma (Poole, UK). IL-6 was purchased from Pepro Tech (NJ, USA). The primary antibodies against total STAT3, COX-2, and β-actin and streptavidin/peroxidase for immunochemical staining were purchased from BIOS China, and the antibody against phosphorylated tyrosine705 STAT3 (p-tyr-705 STAT3) was purchased from Cell Signaling Technology (Massachusetts, USA). The anti-C/EBPβ antibody was purchased from Santa Cruz (California, USA). Cell culture dishes were purchased from NECU (Denmark). IL-6 was dissolved in acetic acid to a stock concentration of 1 μg/ml, and AG490 was dissolved in DMSO to a stock concentration of 100 mM/L. Both stock solutions were stored at −20°C for further use. For all experiments, the optimal working concentrations of the tested reagents were prepared by diluting with RPMI-1640 medium.

### SSd and Its Preparation

The SSd extract (purity ≥ 95%) from *Bupleurum falcatnum* was purchased from Sigma (Poole, UK). For all experiments, a stock solution was prepared by dissolving SSd into DMSO to a concentration of 10 mg/ml and stored at −20°C. The final concentrations of the tested compound were prepared by diluting the stock solution with DMEM. The final concentration of DMSO was less than 0.1%.

### Cell Lines and Cell Culture

The human HCC cell line SMMC-7721 was a kind gift from Professor Chen Wei (the First Affiliated Hospital of Xi’an Jiaotong University), and the human HCC HepG2 cell line was kindly provided by Urology Institute of Xi’an Jiaotong University. The identity and authentication of both cell lines used was confirmed by relevant authorized STR profile reports. Both SMMC-7721 and HepG2 cells were cultured as described previously and have been used extensively to study liver cancer (He et al., 2014).

### Cell Proliferation Assay

The effect of SSd on cell proliferation was tested using the MTT assay. The cells were plated in 96-well plates at a density of 5 × 10^3^ cells per well and were allowed to grow to 70% conﬂuence. After 24 h, the cells were separated into four treatment groups and treated with different concentrations of SSd (2.5, 5.0, 10.0, and 15.0 μg/ml). After 24-, 48-, and 72-h incubation, freshly prepared MTT test solution was added to each well. After a 4-h incubation period, the supernatant was discarded and 150 μl DMSO was added to dissolve the crystals. All analyses were performed in biological triplicates. The absorbance was measured using an ELISA reader at a wavelength of 490 nm. The proliferation inhibition rate (PIR%) was calculated using the following formula: (PIR%) = (control well A490 − experimental well A490)/control well A490 × 100%.

### Apoptosis Assay

Apoptosis analysis of both SMMC-7721 and HepG2 cells was conducted using the Annexin V-FITC Apoptosis Detection Kit according to the manufacturer’s instructions (Invitrogen, CA, USA). Briefly, cells (2 × 10^6^ cells/dish) were seeded into six-well plates. Following 24-h treatment with and without SSd (5.0 mg/ml), cells were removed from the plates using trypsin, washed with ice-cold PBS twice, and harvested. The cells were then resuspended to approximately 1 × 10^6^ cells/ml and stained with Annexin V-APC and propidium iodine according to the manufacturer’s instructions (KeyGEN BioTECH). Annexin V-APC/PI binding was analyzed by flow cytometry using a BD FACSCalibur system. Each histogram was constructed with the data from at least 5,000 events. All the samples were analyzed in triplicate.

### Immunocytochemistry

Immunocytochemical staining was performed to assess the expression of COX-2, p-STAT3, and STAT3 proteins in SMMC-7721 cells. Cells were plated on coverslips in 24-well cell culture plates at a cell density of 10 × 10^4^ cells/well. When the cells reached 60–70% confluency, they were separated into different treatment groups. The staining was performed on the coverslips obtained from each of the treatment groups. Immunocytochemistry S-P (Streptavidin/Peroxidase) methods were used according to the manufacturer’s instructions. Briefly, the slides were placed into 0.1% Triton-X 100 for 5 min and incubated for 15 min in 3% hydrogen peroxide at room temperature. After washing with PBS (pH 7.4), the slides were blocked by blocking reagent (normal goat serum) for 15 min at room temperature. The slides were incubated with primary antibody (rabbit anti-human) at 4°C overnight in a humidity chamber. Slides were washed with PBS and then incubated with goat biotinylated anti-rabbit immunoglobulin G for 10 min and then incubated with streptavidin/horseradish peroxidase for 10 min at 37°C. Finally, the slides were incubated with DAB working solution (Tiangen, China) for 5 min and counterstained with hematoxylin (nuclear counterstain) after they were washed with PBS. As a negative control, sections were treated with PBS with the omission of the primary antibody.

The images were quantitatively analyzed using ImagePro Plus 7.1 software (Media Cybernetics, Silver Spring, MD) as described in previous studies (Fang et al., 2013; Liu et al., 2014). The threshold for positive staining was defined by a pathologist who was blinded to the treatment. This threshold was used to analyze all of the subsequent samples. The results, which represent the average positive staining above the threshold for individual sections, were expressed as image units. The mean of these values represents the amount of staining per treatment group used for subsequent statistical comparison. The reading from the control group was set to 1, and the values for the others were derived from actual readings divided by the control reading.

### Western Blotting Analysis

Both SMMC-7721 and HepG2 cells were seeded into six-well plates (2.5 × 10^5^/well). After 24 h, the cells were divided into different groups and treated with vehicle (Control group), IL-6 (25 ng/ml) only, IL-6 + SSd (2.5, 5.0, and 10.0 µl/ml), or IL-6 + AG90 (10, 50, and 100 µmol/L), by adding the indicated drug concentrations directly into the cell culture medium. The next day, tumor cells were lysed in lysis buffer and centrifuged at 12,000×*g* for 15 min. Protein concentrations were determined using a Pierce™ BCA Protein Assay Kit (Thermo Fisher Scientific) following the manufacturer’s instructions. The protein was separated by 10% SDS polyacrylamide gel electrophoresis and then transferred to a polyvinylidene fluoride membrane. After blocking for 1 h with 5% milk in tris-buffered saline and tween 20, the primary antibodies [total STAT3 (1:200), p-tyr-705 STAT3 (1:1,000), C/EBPβ (1:1,000), COX-2 (1:1,000), and β-actin (1:300)] were added and incubated at 4˚C overnight. After incubation with secondary antibodies, horseradish peroxidase-conjugated secondary antibody (1:3,000), membranes were visualized with ECL (Santa Cruz, CA) detection. Protein bands were scanned using Odyssey bands scanner (S/N ODY-2792 model: 9120). The intensities of the bands were analyzed using Bandscan software.

### Quantitative Reverse Transcriptase-PCR

Quantitative reverse transcriptase-PCR (qRT-PCR) was conducted to assess the expression of mRNA for COX-2, STAT3, and C/EBPβ in both SMMC-7721 and HepG2 cells after treatment with SSd at various concentrations or addition of AG490. Cells were first seeded into 6-cm dishes (2 × 10^6^ cells/dish). After 24-h incubation, cells in treatment groups (groups 2–5) were then treated with IL-6 at 25 ng/ml plus SSd (0, 2.5, 5.0, and 10.0 µg/ml) or JAK2 kinase inhibitor AG490 (0, 10, 50, and 100 µmol/L) for a further 24 h. The total RNA in cells in all treatment groups was extracted using TRIzol reagent (Invitrogen, CA, USA). RNA integrity was confirmed by absorption at 260 and 280 nm using a spectrophotometer (Beckman Coulter Du® 800, CA, USA). cDNA was synthesized using Transcript High Fidelity cDNA Synthesis Kit (Fermentas). The primer sequences for target genes of COX-2, STAT3, C/EBPβ, and β-actin are detailed in Table 1. Using the Light Cycler 480 SYBR Green I Master Mix (Roche), qRT-PCR was performed according to the qRT-PCR manufacturer’s protocol (Invitrogen, CA, USA). Melting curve detection was used to analyze the specificity of qRT-PCR products. The expression of mRNAs was analyzed by Mx Pro QPCR software version 3.0, and the housekeeping gene, β-actin, was used as an internal control to normalize variations in the integrity and total amount of cDNA. Data are expressed as relative expression as described by Livak and Schmittgen (Livak and Schmittgen, 2001).

**Table 1:** Primers used for RT-qPCR.

Target gene	Forward primer (5′→3′)	Reverse primer (5′→3′)
COX-2	AGTATCACAGGCTTCCATTGACCAG	CCACAGCATCGATGTCACCATAG
STAT3	GGCTTCTCCTTCTGGGTCTGG	TCTTACCGCTGATGTCCTTCTCC
C/EBPβ	GTTCATGCAACGCCTGGTG	AAGCAGTCCGCCTCGTAGTAGAAG
β-actin	ATCGTGCGTGACATTAAGGAGAAG	AGGAAGGAAGGCTGGAAGAGTG

### Luciferase Reporter Assay

Bioinformatic analysis (JASPAR (http://jaspar.genereg.net/) was used to predict binding sites between transcription factors and gene promoters. HepG2 cells seeded in 96-well plates were cultured for 24 h, reaching 60–80% confluency before transfection. The luciferase reporter vector, the wild-type (WT), or mutant (Mut) (GeneChem, China) together with pcDNA3.1 plasmid (GeneChem, China) were co-transfected using the Lipofectamine 2000 reagent (Invitrogen, Carlsbad, CA). At 48 h post-transfection, the Dual Luciferase Assay Kit (Promega) was used to examine the luciferase activity according to the manufacturer’s instructions. Renilla luciferase activity was used as a control.

### Cell Transfection Assay

All the small interfering RNA (siRNA) sequences targeting STAT3 (Genepharma, Shanghai, China) have been listed in Table 2. STAT3 knockdown was performed by transfecting STAT3-siRNA#1-3. Transfection assays were conducted when the cells reached approximately 60–80% confluency according to the manufacturer’s instructions. Total RNA from cells was extracted 48 h post-transfection.

**Table 2:** siRNA sequences used in the present study.

Genes	Sense 5′-3′	Antisense5′-3′
STAT3-siRNA1-398	CCACUUUGGUGUUUCAUAATT	UUAUGAAACACCAAAGUGGTT
STAT3-siRNA2-978	GCAACAGAUUGCCUGCAUUTT	AAUGCAGGCAAUCUGUUGCTT
STAT3-siRNA3-1070	CCCGUCAACAAAUUAAGAATT	UUCUUAAUUUGUUGACGGGTT

### Statistical Analysis

All statistical analysis was performed using SPSS package version 24.0. The results were expressed as means ±SD, as indicated. All treatments were arranged in a randomized block design with three replicates. Analysis of variance was used for comparison among different treatment groups. The difference was considered statistically signiﬁcant when *P* < 0.05.

## Results

### Inhibitory Effect of SSd on Cancer Cell Proliferation

**Figure 1:**
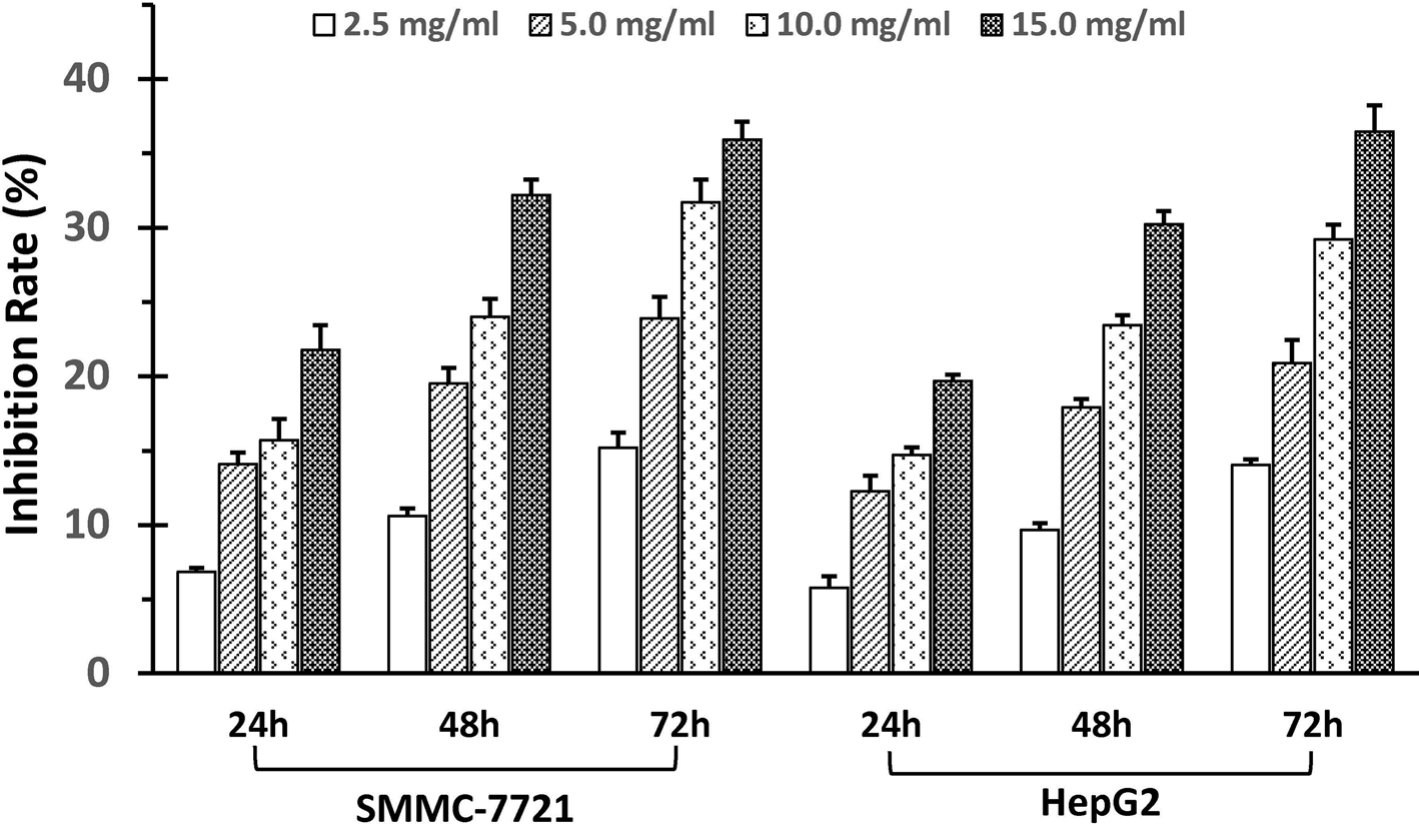
SSd inhibited proliferation of SMMC-7721 and HepG2 cells. Adherent liver cancer cells (SMMC-7721 and HepH2) were seeded in 96-well plates (5 × 10^3^cells/well) and incubated with different concentrations of SSd ranging from 2.5 to 15 µg/ml, and time intervals ranging from 24 to 72 h, as indicated on the histograms. Cell proliferation was determined by the MTT assay. Data were expressed as mean ± SD.

The SSd antiproliferative effects in human HCC cancer cell lines, SMMC-7721 and HepG2, were demonstrated in a dose- and time-dependent manner using the MTT proliferation assay. As illustrated in **Figure 1**, the degree of inhibition was concomitant with an increase in SSd dosage, and the significance (*P* > 0.05) was demonstrated in all treatment groups compared to control (vehicle).

### SSd Induced Apoptosis

**Figure 2:**
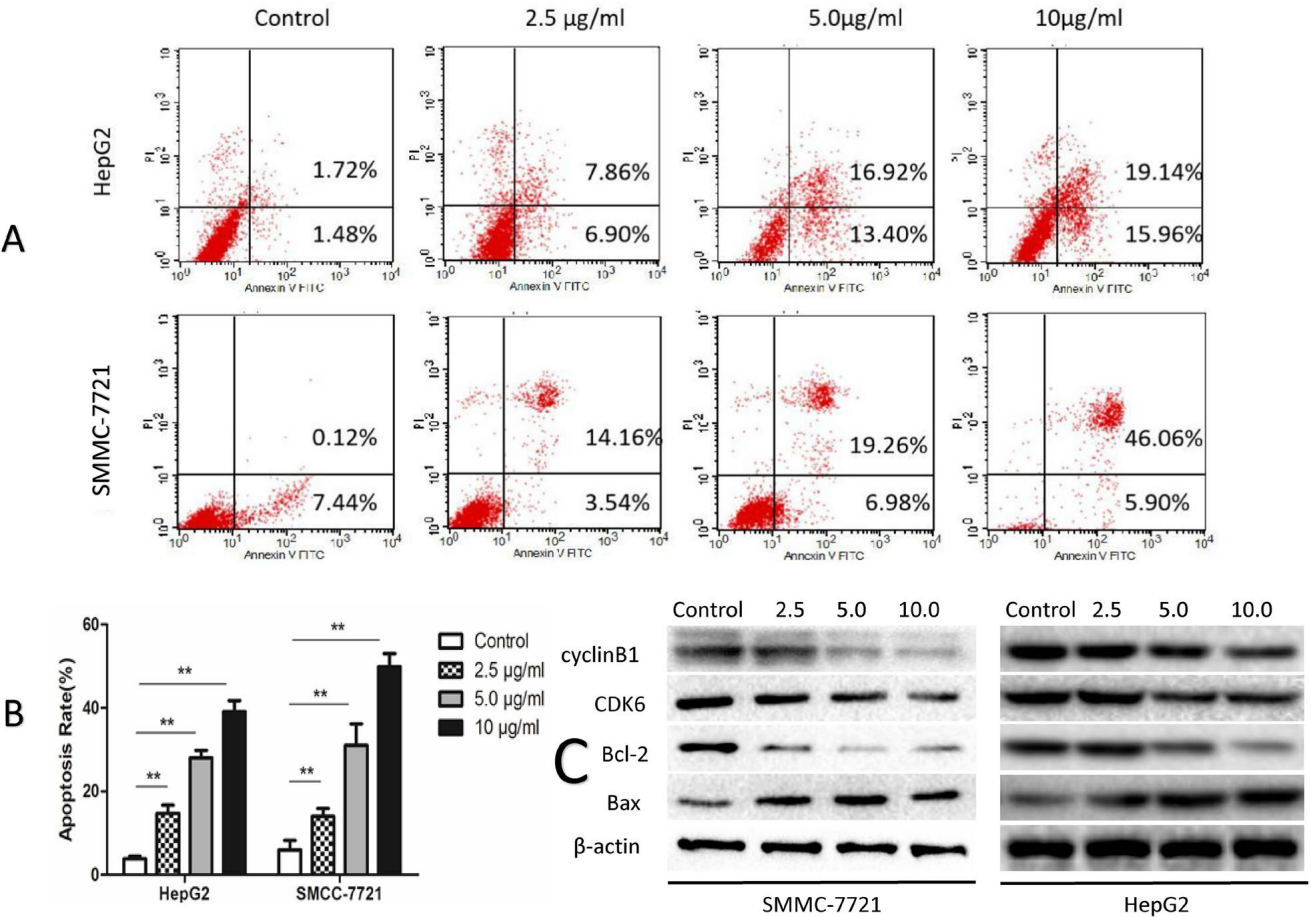
SSd increased apoptosis in SMMC-7721 and HepG2 cells. **(A)** The tumor cells were treated with SSd at various concentrations or vehicle (control) and analyzed by flow cytometric analysis. **(B)** Apoptosis rates in all treatment groups are presented as a histogram (data were expressed as mean ± SD). **(C)** Western blots of Bax (a pro-apoptotic protein), CDK6, Bcl-2, and cyclin B1. β-Actin was used as a loading control

The degree of apoptosis was analyzed by flow cytometry in all treatment groups and compared to the control groups in both SMMC-7721 and HepG2 cells (**Figure 2A**). Twenty-four hours post-treatment with SSd, the percentages of apoptotic cells were significantly increased in both cell types in a dose-dependent fashion compared to controls (*P* < 0.05 or 0.01) (**Figure 2B**). At the protein level, SSd treatment also resulted in an increase in the pro-apoptotic protein Bax and a decrease in the anti-apoptotic protein Bcl-2. The high expression of CDK6 (a key protein kinase, which activates cell proliferation) and cyclin B1 was inhibited by SSd in both SMMC-7721 and HepG2 cells (**Figure 2C**).

### SSd Suppressed Protein Expression of p-STAT3 and COX-2

**Figure 3:**
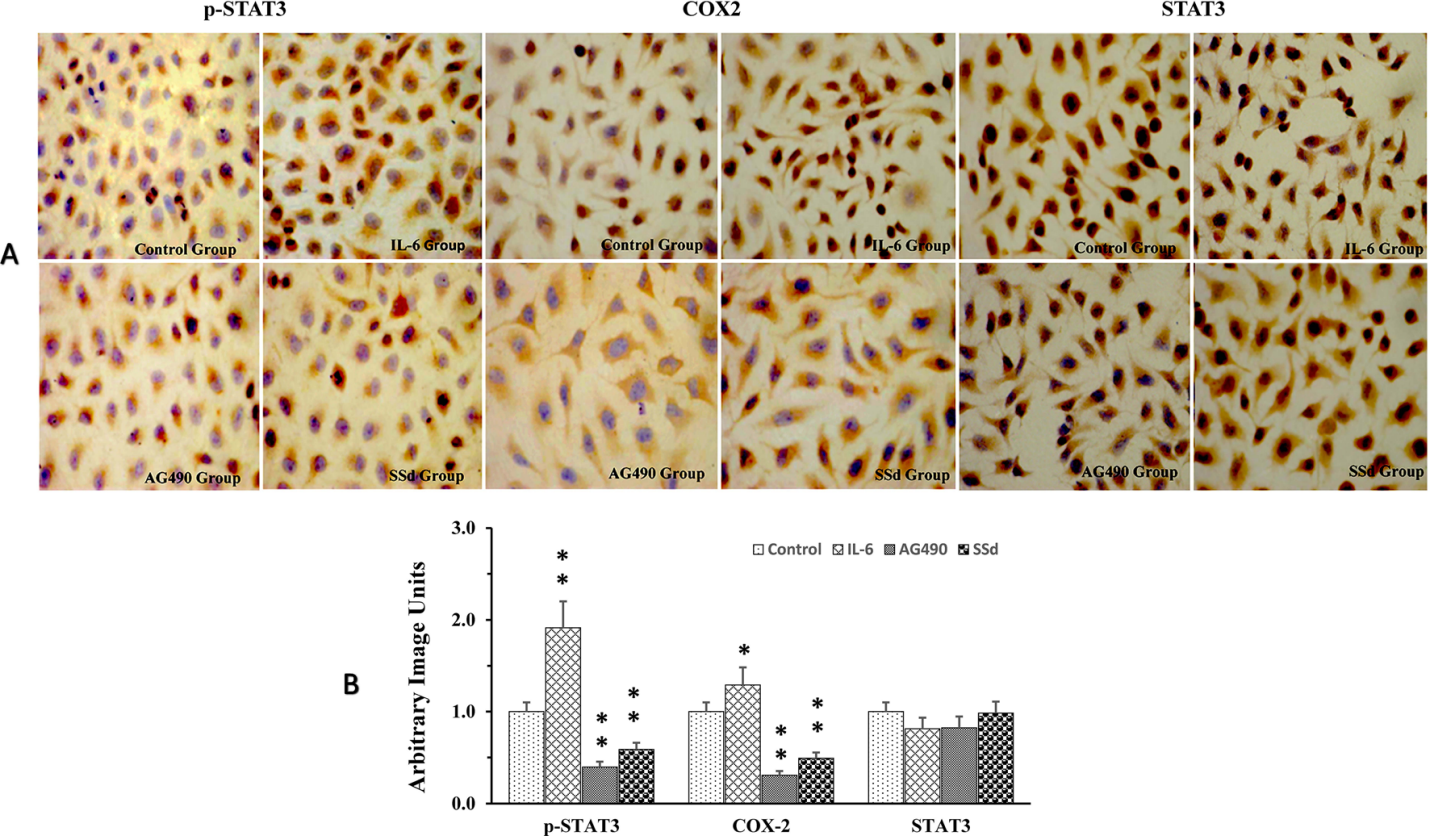
Alteration of p-STAT3, COX-2, and STAT3 expression post-treatment with SSd. **(A)** Representative images of immunocytochemical staining of SMMC-7721 cells pre- and post-treatment with AG490, SSd, and IL-6. Control group: Cells treated with PBS; IL-6 group: Cells treated with IL-6 (25 ng/ml); AG90 group: Cells treated with IL-6 (25 ng/ml) + AG490 (25 µmol/L); and SSd group: Cells treated with IL-6 (25 ng/ml) + SSd (5 µg/ml). DAB used as chromogen; original magnification ×200: nuclear immunoreactivity. **(B)** Results from quantitative analysis of images using ImagePro Plus 7.1 software and data were expressed as arbitrary image units. **P < 0.01, *P < 0.05 compared to the control. Nucleus was stained using hematoxylin (blue), antibody staining (brown).

We utilized immunohistochemical staining to determine the expression and localization of STAT3, p-Stat3, and COX-2 in tumor cells. Total STAT3, COX2, and p-tyr-705 STAT3 strongly stained in the nuclear compartment (brown staining) in the control groups. Immunocytochemistry quantitation, using ImagePro Plus 7.1 software, showed significant increase in p-tyr-705 STAT3 and COX-2 when the cells were exposed to IL-6; however, no significant changes were observed in total STAT3 with IL6 treatment. Both AG490 and SSd effectively inhibited the expression of p-tyr-705 STAT3 and COX-2 (**Figure 3A and B**). However, the expression of total STAT3 showed no difference between the control group and the SSd group (**Figure 3A and B**). Both AG490 and SSd effectively inhibited the expression of p-tyr-705 STAT3 and COX-2 (**Figure 3A and B**). However, the expression of total STAT3 showed no difference between the control group and the SSd group (**Figure 3A and B**). The inhibition of p-STAT3 and COX-2 expression by both SSd (5 μg/ml) and AG490 (25 μmol/L) was statistically significant (*P* < 0.01). Interestingly, while treatments with AG490 and SSd significantly decreased nuclear COX2, a slight increase in COX2 expression was present in the cytoplasmic compartment.

### SSd Inhibited p-STAT3, C/EBPβ, and COX-2 Protein

**Figure 4:**
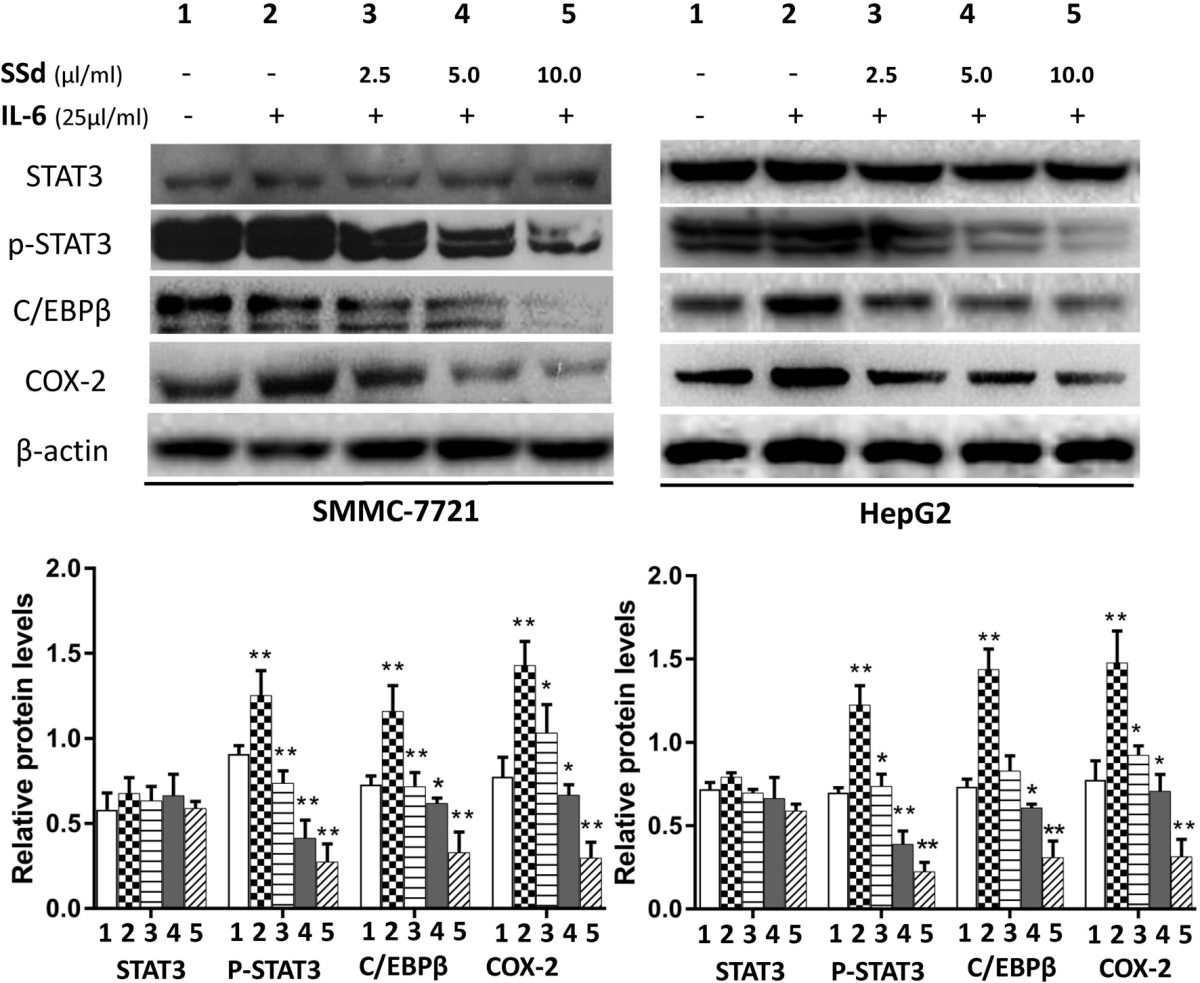
Protein expression of total STAT3, p-STAT3, C/EBP**β**, and COX-2 following treatment with SSd. SMMC-7721 and HepG2 cells were seeded into six-well plates (2.5 × 10^5^/well). After 24-h culture in RPMI-1640 medium, the cells were divided into five groups and treated with SSd in the following conditions: 1) Control group cells received no drug treatment; 2) cells treated with IL-6 (25 ng/ml) only; 3) cells treated with IL-6 (25 ng/ml) + SSd (2.5 µg/ml); 4) cells treated with IL-6 (25 ng/ml) + SSd (5.0 µg/ml); 5) cells treated with IL-6 (25 ng/ml) + SSd (10.0 µg/ml). Representative Western blot of results are shown in upper panels. For the quantitation of Western blots, protein expression was normalized to β-actin levels in each lane and expressed relative to levels in normal cells. The data are presented as the mean ± SD of three separate experiments. *P < 0.05 and **P < 0.01 when compared with group 2, where cells were treated with IL-6 only.

**Figure 5:**
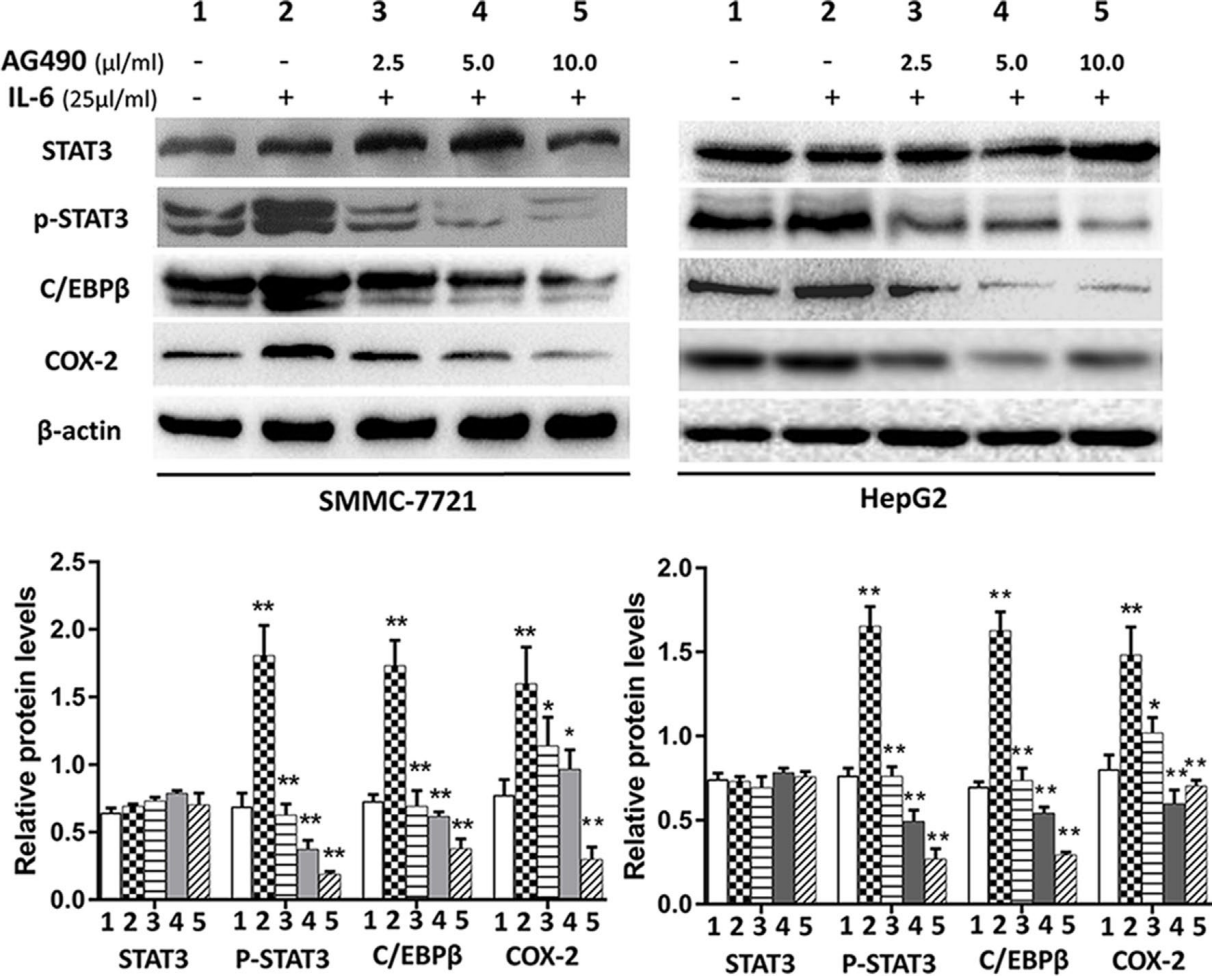
Protein expression of total STAT3, p-tyr-705 STAT3, C/EBPβ and COX-2 following treatment with AG490 (JAK2 inhibitor). Cell culture conditions are as described in **Figure 4**. Tumor cells (HepG2 and SMMC-7721) were divided into different groups and treated with different concentrations of AG490 as follows: 1) control group: cells received no drug treatment; 2) cells treated with IL-6 (25 ng/ml) only; 3) cells treated with IL-6 (25 ng/ml) + AG490 (10 µmol/L); 4) cells treated with IL-6 (25 ng/ml) + AG490 (50 µmol/L); 5) cells treated with IL-6 (25 ng/ml) + AG490 (100 µmol/L).

STAT3 and C/EBPβ are key signaling molecules involved in carcinogenesis of HCC. Here, we determined the effects of SSd on the activation of STAT3 by measuring the level of p-STAT3 (tyr 705) in the total protein extracts. It is well known that the transcription factor CCAAT/enhancer-binding protein (C/EBPβ) plays a key role in regulating COX-2 gene expression (Thomas et al., 2000; Suh et al., 2006). Therefore, we determined whether C/EBPβ was also an important target for SSd in these tumor cells. The representative images of Western blotting results from all treatment groups are presented in **Figures 4** and **5**. As shown in **Figures 4** and **5**, IT-6 (25 ng/ml) treatment resulted in nuclear translocation and phosphorylation of STAT3 in both cell types. The protein expression of C/EBPβ, p-tyr-705 STAT3, and COX-2 was significantly higher compared to untreated cells (*P* < 0.01). Following the addition of SSd at various concentrations in cell culture, the protein expression of all C/EBP-β, p-tyr-705 STAT3, and COX-2 was significantly inhibited (*P* < 0.01) in both SMMC-7721 and HepG2 cells, and the inhibition was demonstrated in a dose-dependent manner (**Figure 4**). The observed inhibition of protein expression of C/EBPβ, p-tyr-705 STAT3, and COX-2 by AG490 (**Figure 5**) was similar to that demonstrated by SSd. The protein level of total STAT3 did not vary significantly among the five treatment groups.

### Effects of SSd on the Expression of mRNA for STAT3, C/EBPβ, and COX-2

**Figure 6:**
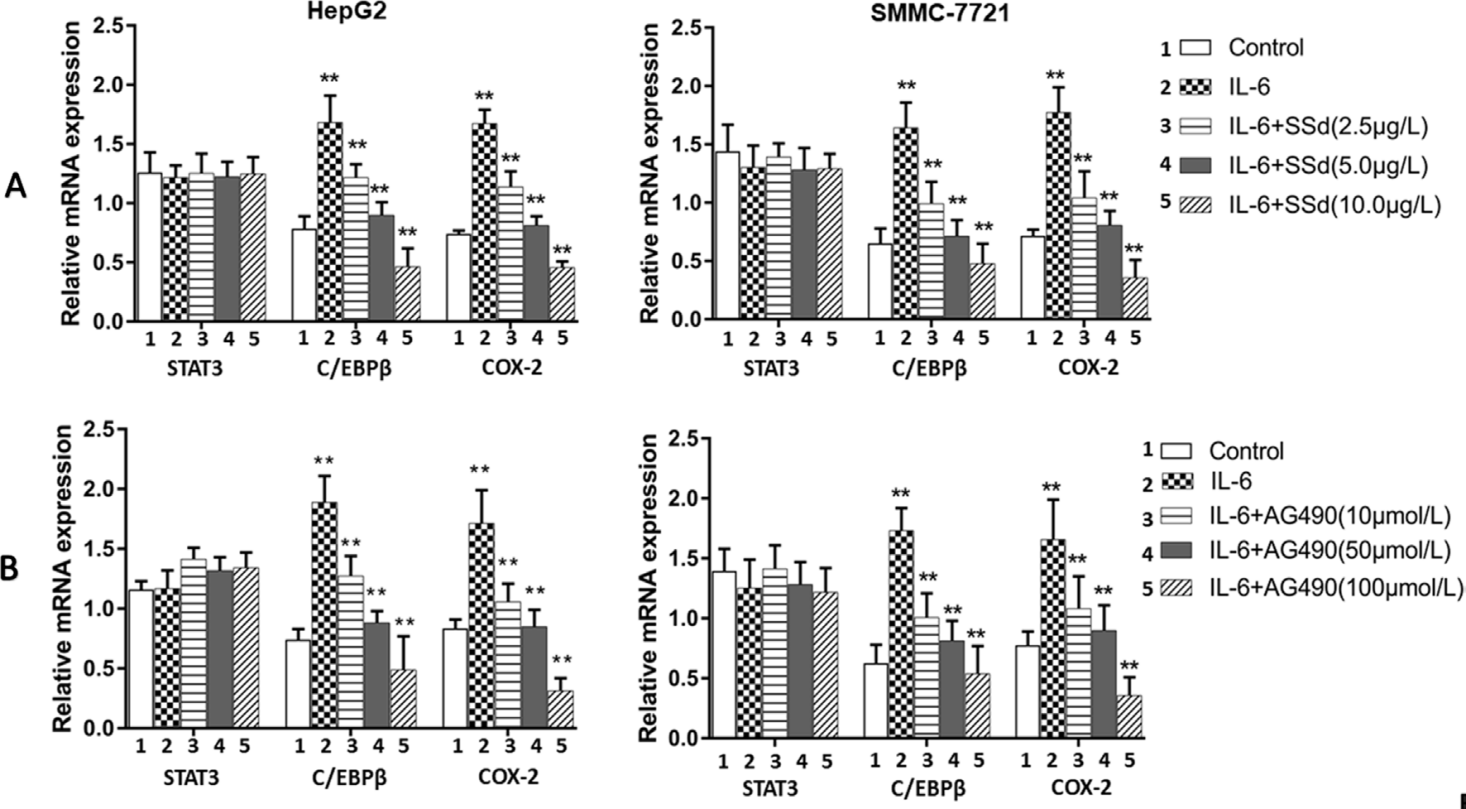
Expression of mRNA for total STAT3, C/EBPβ, and COX-2 as determined by qRT-PCR. **(A)** SSd treated cells. Upper panel: Tumor cells (HepG2 and SMMC-7721) were divided into five groups and treated with different concentrations of SSd as follows: 1) control group: cells received no drug treatment; 2) cells treated with IL-6 (25 ng/ml) only; 3) cells treated with IL-6 (25 ng/ml) + SSd (2.5 µg/ml); 4) cells treated with IL-6 (25 ng/ml) + SSd (5.0 µg/ml); 5) cells treated with IL-6 (25 ng/ml) + SSd (10.0 µg/ml). **(B)** AG490 treated cells. Lower panel: Both HepG2 and SMMC-7721 cells were divided into identical five groups and treated AG490 as various concentrations as follows: 1) control group: cells received no drug treatment; 2) cells treated with IL-6 (25 ng/ml) only; 3) cells treated with IL-6 (25 ng/ml) + AG490 (10 µmol/L); 4) cells treated with IL-6 (25 ng/ml) + AG490 (50 µmol/L); 5) cells treated with IL-6 (25 ng/ml) + AG490 (100 µmol/L). Data are expressed as relative expression using the ΔΔCq method. **P < 0.01 and *P < 0.05 compared with group 2 (cells treated with 25 ng/ml IL-6).

The mRNA expression of the target genes in the tumor cells was analyzed by qRT-PCR. The expression of mRNA for COX-2 and C/EBPβ was significantly higher in the IL-6 treated group compared to the control group (*P* < 0.01) (shown in **Figure 6**). However, the increased mRNA expression was abrogated by SSd and AG490, and the inhibition was observed in a dose-dependent manner. When compared with cells treated with IL-6, the mRNA expression for both COX-2 and C/EBPβ was significantly abrogated by both SSd and Ag490 (*P* < 0.05 and 0.01), and the observed inhibition by SSd was similar to that observed by AG490 treatment. STAT3 expression showed no significant difference in all treatment groups tested.

### STAT3/C/EBPβ Signaling Pathway Regulated the Expression of COX2 in HCC Cells

**Figure 7:**
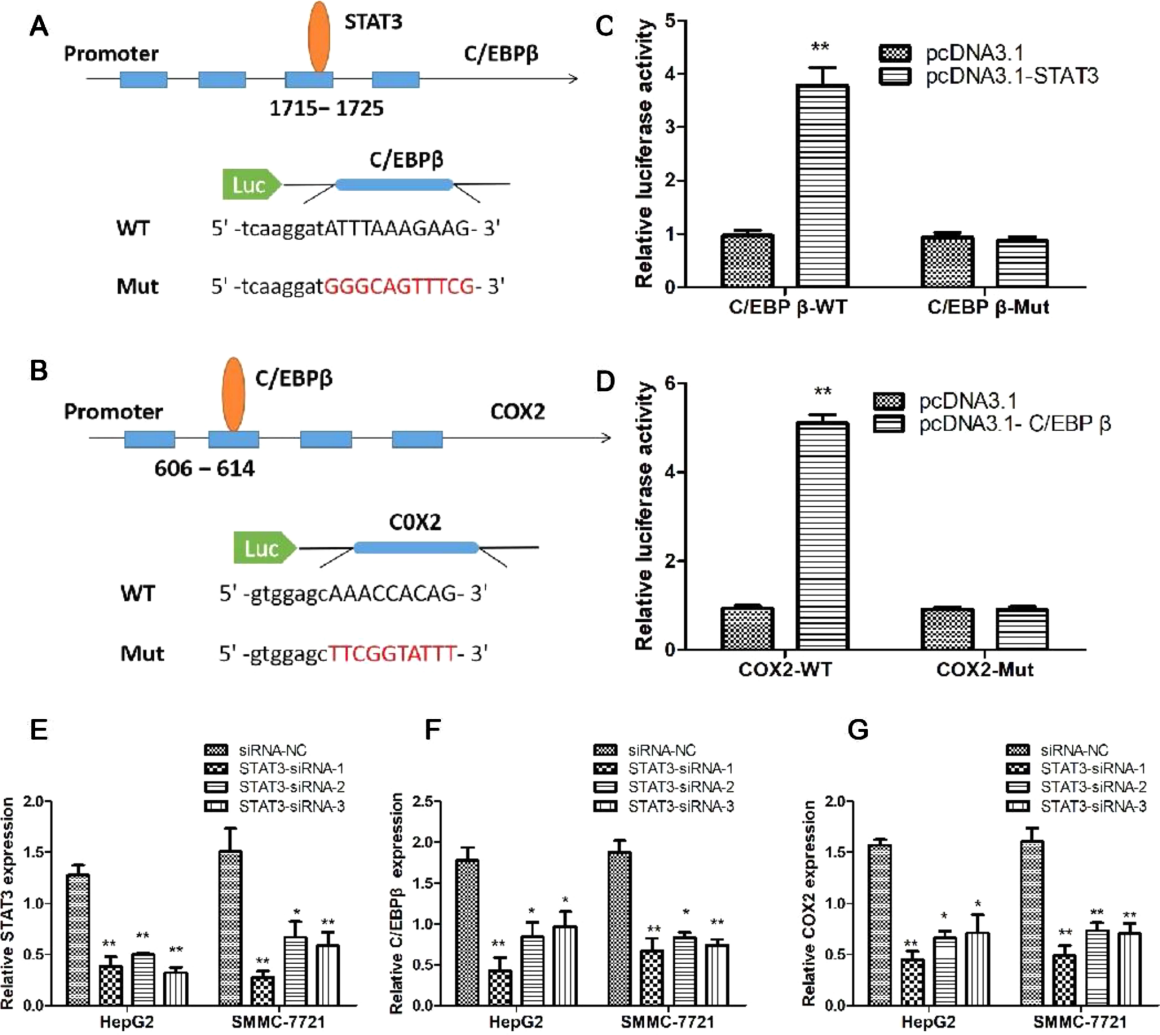
STAT3/C/EBPβ signaling pathway regulates the expression of COX2 in HCC cells. Schematic illustrating the STAT3 binding sites at the promoter of C/EBPβ **(A)** and the C/EBPβ binding sites at the promoter of COX2 **(B)**. **(C)** Luciferase reporter assay was applied to verify the targeted binding effect between STAT3 and C/EBPβ. **P < 0.01. **(D)** Luciferase reporter assay was applied to verify the targeted binding effect between C/EBPβ and COX2. **P < 0.01. **(E)** qRT-PCR analysis of STAT3 expression following transfected HepG2 and SMMC-7721 cells with STAT3-siRNA1-3. *P < 0.05, **P < 0.01. **(F)** qRT-PCR analysis of C/EBPβ expression following transfected HepG2 and SMMC-7721 cells with STAT3-siRNA1-3. *P < 0.05, **P < 0.01. **(G)** qRT-PCR analysis of COX2 expression following transfected HepG2 and SMMC-7721 cells with STAT3-siRNA1-3. *P < 0.05, **P < 0.01

In order to verify the regulatory mechanisms of STAT3/C/EBPβ/COX2 signaling pathway, we used the JASPAR program to predict the binding sites between these genes. The results suggested that STAT3 has a potential binding site on the C/EBPβ promoter; in addition, C/EBPβ has a potential binding site on the COX2 promoter. To verify the validity of the binding sites between genes, the luciferase reporter vectors were constructed for C/EBPβ and COX2 promoters (**Figure 7A and B**). The luciferase reporter assay results showed co-transfection of cells with C/EBPβ-WT vector, and pcDNA3.1-STAT3 significantly increased luciferase reporter activity; however, C/EBPβ-Mut in STAT3’s putative targeting sites did not result in these effects (**Figure 7B**). Similarly, co-transfection of cells with COX2-WT vector and pcDNA3.1-C/EBPβ significantly increased luciferase reporter activity; however, COX2-Mut in C/EBPβ’s putative targeting sites did not result in these effects (**Figure 7D**). In order to investigate the regulation of STAT3 on C/EBPβ and COX2 expression, three STAT3-specific small interfering RNAs (siRNA1-3) and a negative control (siRNA-NC) were transfected into HepG2 and SMMC-7721 cells to evaluate the inhibition efficiency of STAT3. As shown in **Figure 7E**, STAT3-siRNA1-3 produced the greatest reduction in endogenous STAT3 expression. Meanwhile, compared with the control group, interfering with the expression of STAT3 significantly down-regulated the mRNA levels of C/EBPβ and COX2 (**Figure 7F and G**). These results suggest that STAT3/C/EBPβ signaling positively regulates the expression of COX2 in HCC.

## Discussion

Building on our previous work, which identified the significant role of SSd in COX2 suppression in hepatocarcinogenesis and its chemo-preventative effects in HCC (He et al., 2006; He et al., 2014; Liang et al., 2010; Lu et al., 2012), in this report, we extended our study to show that anti-tumorigenic effects of SSd act through the intermediatory p-STAT3/C/EBPβ signaling pathway to suppress COX-2. SSd effectively inhibited cell proliferation in a dose-dependent manner via regulating apoptosis. Most importantly, we provided evidence to support the signaling pathway from STAT3 to C/EBPβ, and then to COX2, leading to COX2 suppression by SSd, uncovering the upstream regulatory pathway of COX2. This represents a novel mechanism of action for SSd.

Overexpression of COX-2 has been previously reported to induce tumor initiation, progression, and angiogenesis in solid tumors, including liver cancers (Leng et al., 2003; Foster et al., 2007; Guo et al., 2015; Yang et al., 2016; Chen et al., 2017), identifying anti-COX-2 treatment as an important target for liver cancer. Selective COX-2 inhibitors have demonstrated a significant inhibition on the proliferation of HCC cells (Breinig et al., 2007). The commercially available celecoxib, a selective nonsteroidal anti-inflammatory drug (NSAID) COX-2 inhibitor, has been shown to exert its anticarcinogenic effect in the liver and in liver cell lines by inducing apoptosis through the intrinsic apoptotic pathway (Breinig et al., 2007). Treatment of cancer cells with celecoxib led to demonstrated alterations in the relative levels of the Bcl-2 family, pro-apoptotic proteins increased, and anti-apoptotic proteins decreased (Grosch et al., 2006; Jendrossek, 2013). In keeping with these observations, we demonstrated that the natural product SSd significantly suppressed COX-2 protein and mRNA levels (**Figures 4** and **6**). These findings were accompanied by significant inhibition of cell proliferation in both SMMC-7721 and HepG2 cells in a dose- and time-dependent manner. The magnitude of inhibition in both cell lines was similar (**Figure 1**). We further demonstrated that SSd exerted its anti-carcinogenic effect in these cancer cell lines by decreasing the antiapoptotic protein Bcl-2 and increasing the pro-apoptotic protein Bad (**Figure 2**). The antitumorigenic effects of SSd observed have similar properties to celecoxib treatment, suggesting that pro-apoptosis in our study may be initiated through COX-2 inhibition.

There is considerable information on the downstream regulatory network of COX-2 overexpression linking elevated COX-2 expression to carcinogenesis. COX-2 overexpression has been reported to enhance the expression of key oncogenic genes (HB-EGF, Krt23, Pak1, and TNFRSF12A) and signaling cascades (AKT, STK33, and MTOR pathway), which contribute to the initiation and progression of HCC formation (Chen et al., 2017). To date, no study showing how SSd exerts its COX-2 suppression through the upstream regulatory network has been reported. This report described a novel antitumor action of SSd by inhibition of specific intermediatory upstream regulators of COX-2 in HCC.

To elucidate the mechanism by which SSd inhibits COX-2 expression, we analyzed the protein expression of STAT3, p-STAT, C/EBPβ, and COX-2 and mRNA expression for STAT3, C/EBPβ, and COX-2 genes after treatment with SSd at increasing dosage concentrations. We found that at low concentrations, between 2.5 and 10 µg/ml, SSd effectively suppressed both mRNA and protein expression of C/EBPβ and COX-2 (**Figures 4** and **6**). IL-6 effectively stimulated the expression of C/EBPβ and COX-2 and significantly activated STAT3. Considering SSd suppressed the phosphorylation of STAT3 (active form of STAT3), and AG490 exhibited a similar inhibitory profile to that of SSd on STAT3, p-STAT, C/EBPβ, and COX-2, our results suggested a direct association between SSd-induced inhibition of COX-2 with downregulation of C/EBPβ. Furthermore, we used the JASPAR program to predict the binding sites between STAT3, C/EBPβ, and COX2 genes and revealed that STAT3 has a potential binding site on the C/EBPβ promoter and C/EBPβ has a potential binding site on the COX2 promoter. The luciferase reporter assay was used to validate the binding sites between genes in HCC cells. The results suggested that STAT3/C/EBPβ signaling positively regulates the expression of COX2 in HCC cells, providing evidence of the signaling pathway from STAT3 to C/EBPβ and then to COX2.

In agreement with our data, previous studies demonstrated that the transcription factor C/EBPβ, as an upstream regulator of the COX-2 gene, was significantly elevated in various cancer tissues such as colorectal cancer, human ovarian epithelial tumor, gastric carcinoma (Regalo et al., 2006), prostate cancer (Wang et al., 2007), and human HCC (Lu et al., 2012), further confirming an active role for C/EBPβ in tumorigenesis and cancer development. Other studies have found that the activation of C/EBPβ is crucial for the initial induction of COX-2 by growth factors, tumor promoters, cytokines, and other inflammatory mediators in various cell types (Thomas et al., 2000; Wu et al., 2005), supporting the idea that suppression of this pathway, as demonstrated by SSd, may be an important anti-cancer therapy. Overlapping overexpression of C/EBPβ and COX-2 has been observed in gastric carcinomas, suggesting that C/EBPβ has the potential to mediate gastric carcinogenesis via the regulation of COX-2 expression (Regalo et al., 2006). In human prostate tissues, high correlation of C/EBPβ and COX-2 expression was associated with chronic inflammation and prostate cancer development (Wang et al., 2007). Furthermore, anti-inflammatory drugs, such as salicylate, suppressed COX-2 expression via inhibition of C/EBPβ binding to the COX-2 promoter (Cieslik et al., 2002). Our previous study demonstrated a correlation between C/EBPβ overexpression and COX-2 overexpression in human HCC tissue (Liang et al., 2010). Collectively, these studies provide further support for our present findings, which show that activation of the C/EBPβ and COX-2 pathway plays a vital role in carcinogenesis.

In conclusion, our study demonstrates that the antitumorigenic effects of SSd on HCC cells are a consequence of the suppression of COX-2 expression, which is mediated by downregulation of p-STAT3 via C/EBPβ. Mechanistically, this study supports the idea that SSd blocks phosphorylation and nuclear translocation of STAT3 and then suppresses the expression of C/EBPβ mRNA and protein, leading to the inhibition of COX-2 expression. Linking STAT3, C/EBPβ, and COX-2, this report presents a novel mechanism of action for SSd and advances our understanding of the pharmacological action of SSd in anti-tumorigenicity. Our results also suggests that low doses of SSd, a natural compound extract, shows great potential as a novel alternative chemo-preventive agent for the treatment of HCC.

## Author Contributions

MR participated in all experimental work. MR, EM, YaL, SH, and YiL analyzed the data and drafted, revised, and edited the paper. YiL and SH planned the experiments and applied for research grants. Yal, XZ, XL, and ZZ contributed to several parts of the experiment and revised and edited the manuscript.

## Funding

The study was funded by the National Natural Science Foundation of China (30771895) and Key Program of International Cooperation Project of Shaanxi Province, China (2014KW23-04).

## Conflict of Interest Statement

The authors declare that the research was conducted in the absence of any commercial or financial relationships that could be construed as a potential conflict of interest.
